# Controlled Fabrication of Flower-like Nickel Oxide Hierarchical Structures and Their Application in Water Treatment

**DOI:** 10.3390/molecules17010703

**Published:** 2012-01-12

**Authors:** Feifei Tao, Yongmiao Shen, Linxia Wang

**Affiliations:** Department of Chemistry and Chemical Engineering, Shaoxing University, Shaoxing 312000, China; Email: linxiawanglxw@163.com

**Keywords:** nickel oxide, hierarchical structures, fabrication

## Abstract

Flower-like NiO hierarchical structures with 2–5 μm diameter assembled from nanosheet building blocks have been successfully fabricated via a wet-chemical method combined with thermodecomposition technology. The template-free method is facile and effective in preparing flower-like NiO superstructures in high yield. The intermediate product and final hierarchical structures are characterized by transmission electron microscopy (TEM), scanning electron microscopy (SEM), X-ray diffraction (XRD), Fourier transform IR (FTIR), and thermogravimetric analysis (TGA). The effects of growth temperature and reaction time on the morphologies of the as-prepared structures were investigated by SEM characterization and a possible mechanism for the formation of flower-like NiO is proposed. Based on the nitrogen adsorption and desorption measurements, the BET surface area of the as-obtained sample is 55.7 m^2^/g and the pore-size distribution plot indicates a bimodal mesopore distribution, with pore sizes of ca. 2.6 nm and 7.4 nm, respectively. In comparison with sphere-like and rod-like structures, the flower-like NiO hierarchical structures show an excellent ability to rapidly remove various pollutants when used as adsorbent and photocatalyst in waste-water treatment, which may be attributed to its unique hierarchical and porous surface structures.

## 1. Introduction

Nanoscale materials have been pursued extensively due to their unique physical and chemical properties and promising applications in nano-devices compared to those of their bulk counterparts [1,2]. The morphology, crystallography and size of the nano-structured materials can greatly influence their optical, electronic, magnetic, and catalytic properties [3,4,5,6]. Controlled organization of primary building units with various dimensions into ordered superstructures has been another focus of significant interest for material chemistry and device fabrication [7,8,9,10]. Such a capability is attractive not only in understanding the concept of self-assembly of original building blocks, but also due to the importance in its potential applications [11]. Therefore, the work to obtain these fascinating superstructures needs to be developed to access more novel properties and applications of nanomaterials.

As an important transition metal oxide, NiO is a very promising material and has attracted increasing attention due to its extensive important applications as catalysis [12], electrode materials [13], gas sensors [14] and electrochromic films [15]. Recently, various chemical and physicochemical methods have been employed to produce NiO nanomaterials including nanoparticles [16], nanorods [17], nanowires [18], and nanosheets [19]. The self-assembly of the above nano-sized building blocks to construct NiO hierarchical structures remains of significance to chemists and material researchers, due to their unique structures [20]. Therefore, developing a facile and template-free method to prepare hierarchical NiO structures is of scientific and practical importance.

Here, we report a template-free wet-chemical route to assemble NiO nanosheets into flower-like hierarchical structures combined with a calcination process. The intermediate product first prepared is proved to be a β-Ni(OH)_2_/EG composite. Compared with the adsorption and photocatalytic activities of the sphere-like and rod-like NiO, the excellent and rapid elimination performance of as-prepared NiO samples on various pollutants indicates its potential application in water treatment.

## 2. Results and Discussion

### 2.1. Characterization of Flower-Like Hierarchical Structures

**Figure 1:**
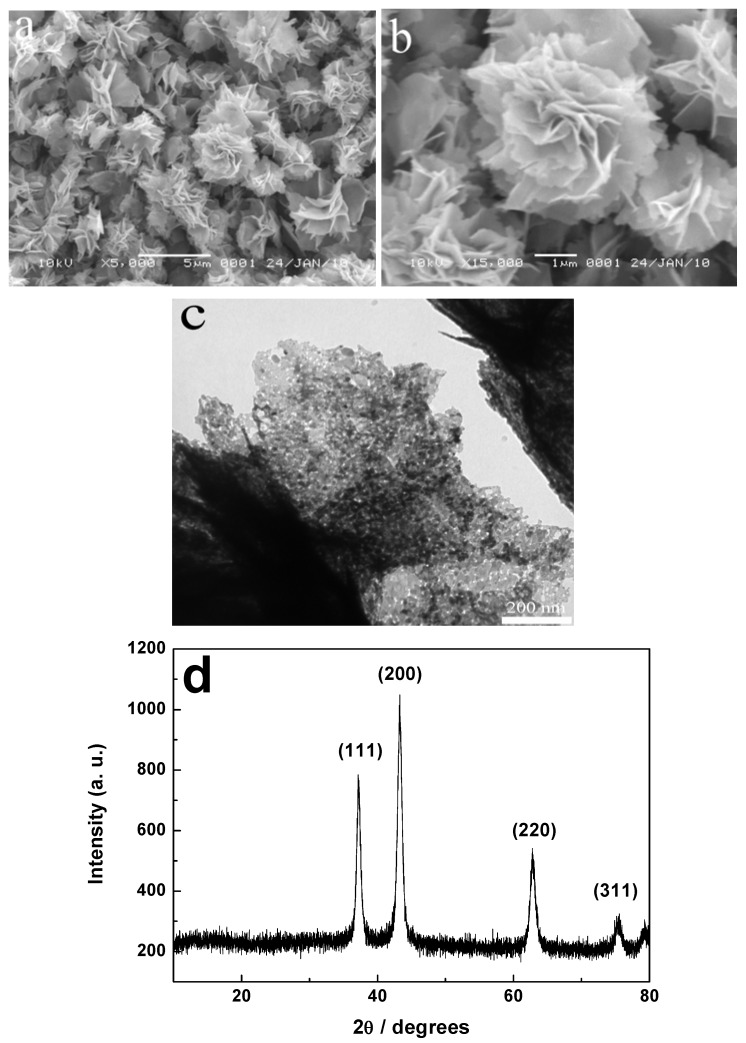
SEM images (**a**,**b**), TEM image (**c**), and XRD pattern (**d**) of the as-prepared products.

Figure 1 shows the SEM and TEM images and XRD pattern of the as-prepared products. In Figure 1a, the SEM image indicates that the flower-like hierarchical structures self-assembled from nanosheets have a large area with a size of 2–5 μm and they hardly have any visible impurities, suggesting a high purity. Image b in Figure 1 further displays the structural feature of interwoven nanosheets with a thickness of about 100 nm as the building blocks to construct the flower-like hierarchical structures. The TEM image in Figure 1c shows the porous features of the nanosheets making up the flower-like hierarchical structures. The XRD pattern in Figure 1d indicates that all recorded peaks can be indexed to face-centered cubic NiO in good agreement with the data of JCPDS file No. 47-1049. No peaks from other phases are found, suggesting the intermediate product was completely converted to NiO.

**Figure 2:**
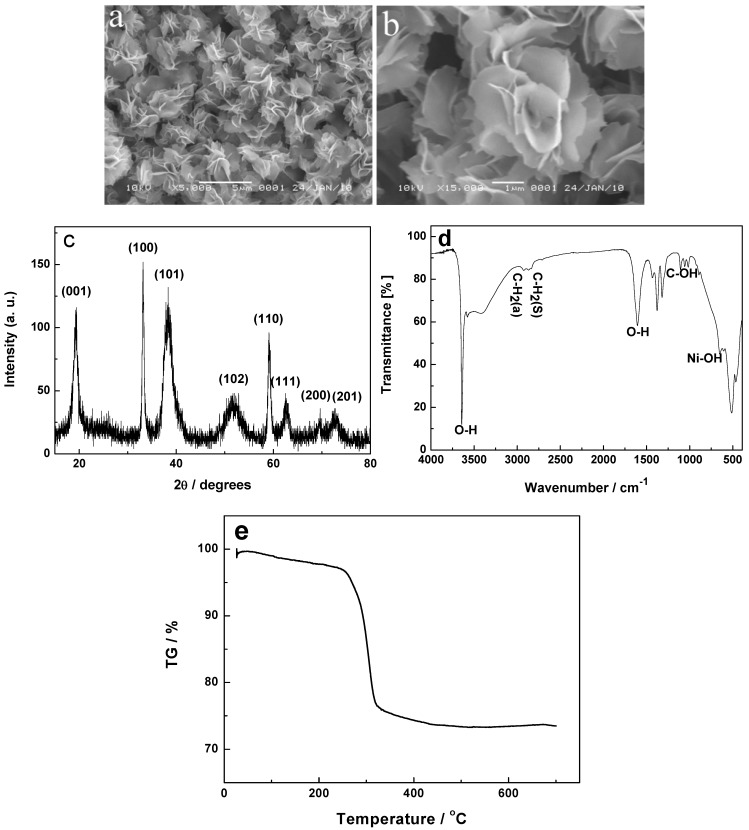
SEM images (**a**,**b**), XRD curve (**c**), FTIR spectrum (**d**), and TGA pattern (**e**) of the intermediate product.

To investigate the effect of calcination on the precursor, the green intermediate products before the annealing treatment are studied by SEM, XRD, FTIR and TGA, as shown in Figure 2. Surprisingly, the SEM images in Figure 2a,b are almost similar to those of the resulting products after the calcination, indicating that the annealing treatment has no influence on the shape of the final products with flower-like hierarchical structures. The XRD pattern (Figure 2c) of the intermediate product shows that all the diffraction peaks are in good agreement with primitive hexagonal-phased β-Ni(OH)_2_ (JCPDS No. 14-0117). As shown in the FTIR spectra (Figure 2d), the absorption at 650 cm^−1^ is due to the Ni-OH bending vibration. The vibrational bands of CH_2_ at 2,927 and 2,859 cm^−1^ as well as the stretching band of C-OH at 1,100–1,025 cm^−1^ derive from EG unit. The TGA data (see Figure 2e) show a total of about 26.6 wt% weight loss up to 500 °C under air, exceeding the calculated value of 19.4 wt% by loss one water molecule and forming NiO, which further illuminates the presence of EG in the intermediate product. Based on the above analysis of XRD, FTIR, and TGA, we can conclude that the green intermediate product with the hierarchical structure is a β-Ni(OH)_2_/EG composite and the EG may intercalate into the interlayered place of β-Ni(OH)_2_ and could not be washed away. After the heating treatment at 350 °C for 2 h, the product is completely converted into NiO, as proven by the XRD pattern in Figure 1d.

### 2.2. Research on the Formation Process of NiO Hierarchical Structures

**Figure 3:**
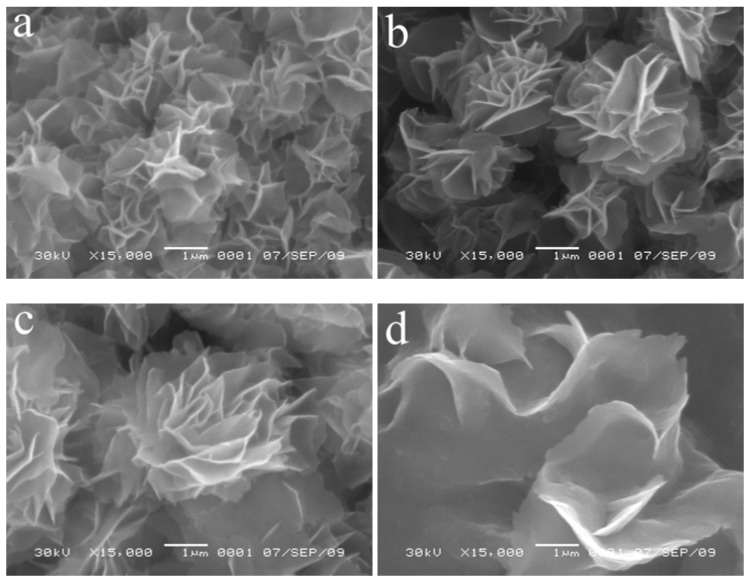
SEM images of the intermediate products obtained at various reaction temperatures: (**a**) 80, (**b**) 100, (**c**) 120, and (**d**) 160 °C.

To gain insight into the mechanism of formation of NiO flower-like hierarchical structures, we only need to investigate that of the intermediate products because the morphology hardly changes after calcination. Based on the experimental results, we find that the reaction temperature plays a crucial role in the formation of flower-like hierarchical structures. In the reaction temperature range of 80–140 °C (Figure 3a–c, Figure 2b), flower-like hierarchical structures assembled from nanosheets with the different sizes could be formed. The sizes of the products increase with the increasing reaction temperatures. When the reaction temperature is higher than 140 °C, only nanosheets are found (Figure 3d). It is suggested that the interaction between the nanosheets responsible for forming flower-like structures could be destroyed at temperatures higher than 140 °C. Also, adjusting the reaction temperatures could selectively control the formation of flower-like hierarchical structures and nanosheets.

**Figure 4:**
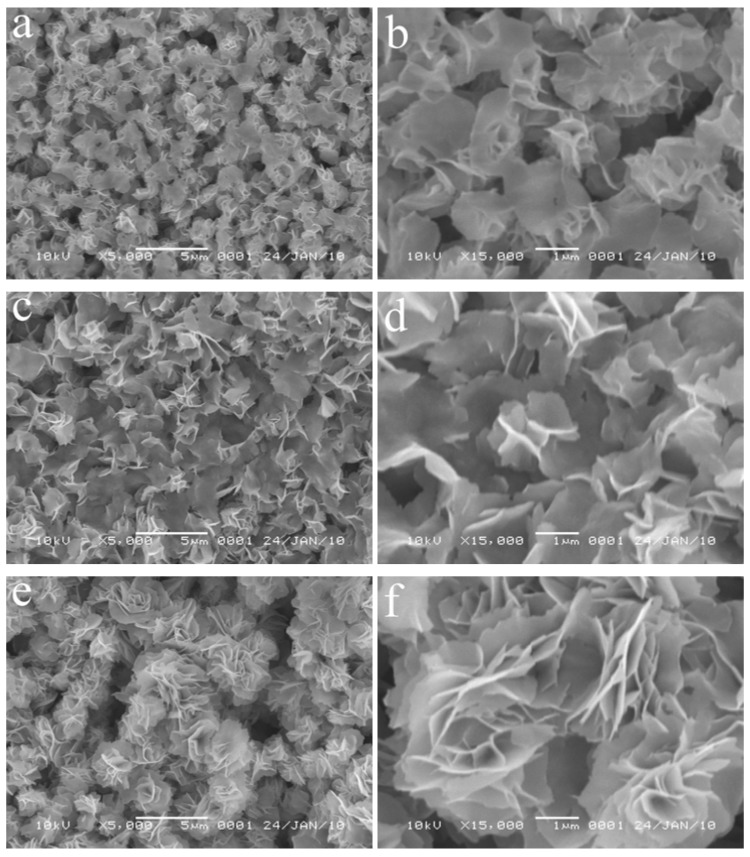
SEM images of the intermediate products reacted at 140 °C for different reaction times: (**a**,**b**) 10 min, (**c**,**d**) 1 h, and (**e**,**f**) 3 h.

Furthermore, time-dependent experiments are carried out at 140 °C and the intermediate products are inspected by SEM. The images are shown in Figure 4, from which the evolution process could be clearly seen. At the beginning of the reaction, first nanosheets are formed and they interweave with each other after reacting for 10 min (images a and b in Figure 4). The nanosheets have a smooth surface and a thickness of about 100 nm. The SEM images of the product reacted for 1 h indicate that as the nanosheets further interweave, the sizes of the nanosheets develop further with the increasing reaction time, while their thickness hardly changes (Figure 4c,d). At the reaction time of 3 h, the nanosheets with unchanged thickness gradually develop in size and further interweave together, and form the preliminary flower-like hierarchical structures, that is the aggregation of flower-like structures (Figure 4e,f). After reaction for 8 h, the aggregations separate and uniform structures with the hierarchical feature are finally formed (Figure 2a,b).

Based on the above experimental results and analysis, we can propose a reasonable mechanism for the formation of the flower-like hierarchical structures assembled from nanosheets. The acetate group from sodium acetate hydrolyzes first, resulting in the formation of OH^−^ ions in the EG solvent. Second, Ni^2+^ ions react with OH^−^ to form sheet-like β-Ni(OH)_2_. Third, Ni(OH)_2_ nanosheets spontaneously attach together to form flower-like aggregations. Finally, the aggregations are gradually divided into uniform flower-like hierarchical assembly constructed by nanosheets, which favours minimization of their surface energy by reducing the surface area.

### 2.3. Nitrogen Physisorption Isotherm of NiO Hierarchical Structures

**Figure 5:**
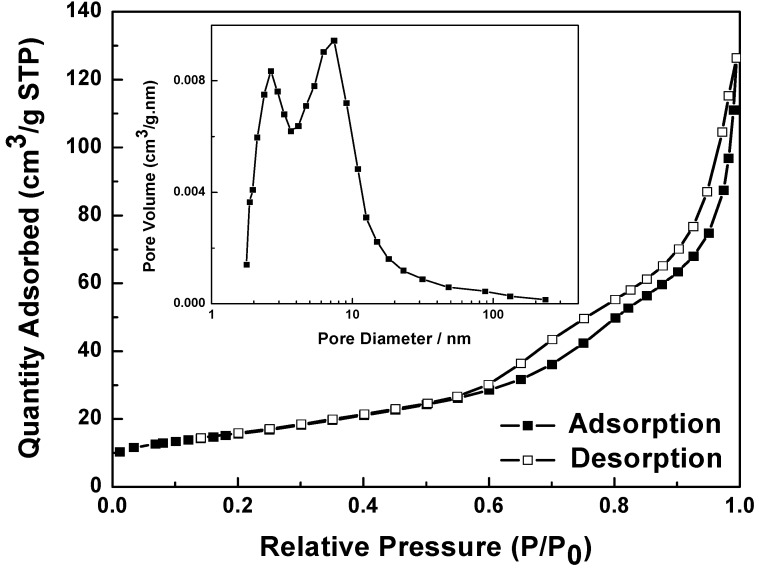
Nitrogen adsorption and desorption of flower-like NiO and BJH pore size distribution derived from the adsorption branch of the isotherm of flower-like NiO in the inset.

The above TEM image (Figure 1c) of calcined hierarchical NiO clearly shows the porous structure, which is further characterized by N_2_ sorption analysis. Figure 5 gives the nitrogen adsorption and desorption isotherms and pore size distribution of flower-like NiO hierarchical structures, revealing that the isotherms belong to type χ [21]. The hysteresis loop appearing in the relative pressure (*p*/*p*_0_) range of 0.5–1 indicates the presence of mesopores in the as-obtained samples, which is associated with the mesopore filling and the monolayer coverage of the mesopore. The BET specific surface area of the NiO hierarchical structure is 55.7 m^2^/g, the pore-size distribution plot (inset in Figure 5) shows a bimodal mesopore distribution, whose pore sizes are about 2.6 and 7.4 nm, obtained by the adsorption branch. This type of porosity would enhance the special surface area and provide an efficient transport pathway for reactants to the interior of the sample, which is beneficial for adsorption and catalytic properties.

### 2.4. Adsorption Properties

**Figure 6:**
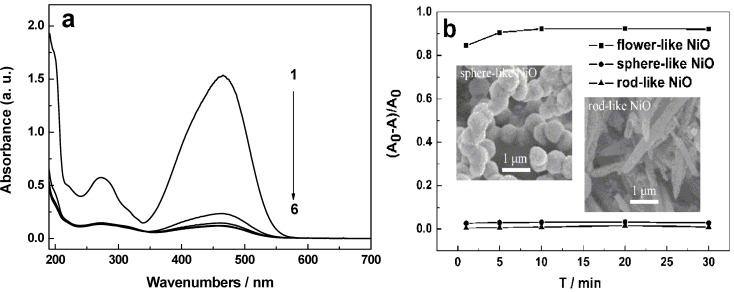
(**a**) Absorption spectra of MO aqueous solutions in the presence of flower-like NiO, 1–6: 0, 1, 5, 10, 20 and 30 min; (**b**) Elimination rates of MO at different intervals in the presence of different adsorbents.

At first we used the flower-like hierarchical NiO constructed by nanosheets as the adsorbent to remove organic pollutants. UV-Vis spectra are applied to demonstrate the adsorption properties of the sample. The characteristic absorption peak at 465 nm of MO is used as a monitored parameter during the adsorption process. Figure 6a shows the absorption spectra of aqueous solutions of MO tested at different intervals in the presence of flower-like NiO hierarchical structures. The absorption intensity of MO at 465 nm decreases gradually with time, indicating the elimination of MO. Figure 6b shows the elimination rates of MO at different intervals in the presence of flower-like, sphere-like and rod-like NiO structures, respectively. Taking the flower-like NiO as the adsorbent, about 85% of MO is eliminated after 1 min, and at the time of 5 min, the adsorption rate exceeds 91%. After that, the elimination rates slowly increase and hardly changes with the increasing time after 10 min. However, the sphere-like and rod-like NiO hardly have any adsorption activity on MO. Such rapid and high elimination rate of flower-like NiO may be due to the porous structure of nanosheets forming the flower-like NiO hierarchical structures, but porous surface structures can hardly been found in the sphere-like and rod-like NiO, as shown in the insets of Figure 6b. In order to further estimate the adsorption performance of flower-like NiO, the elimination rates of various pollutants at the different irradiation times were measured and are listed in Table 1, respectively. The as-prepared flower-like NiO could be widely used to remove various pollutants and the elimination rates of various pollutants in the presence of flower-like NiO were up to 90% and obviously more than those in the presence of sphere-like and rod-like NiO. The unfolded nanosheets and the deep caved pores on the flower-like NiO samples could afford more active sites and enable the architectures to be exposed to the pollutant solutions sufficiently, which favours the adsorption of various pollutants and the diffusion of reactants during the reactions.

**Table 1:** Elimination rates of various pollutions in the presence of flower-like, sphere-like and rod-like NiO samples.

Pollutant	*λ*_max_/nm	NiO	Adsorption time/min	Elimination rates/%
Reactive Red	519	flower-like NiO	10	92.1
		sphere-like NiO	10	3.12
		rod-like NiO	10	1.56
Reactive Brilliant Blue	598	flower-like NiO	5	97.6
		sphere-like NiO	5	5.61
		rod-like NiO	5	2.28
Reactive Black	572	Flower-like NiO	8	96.7
		Sphere-like NiO	8	9.61
		Rod-like NiO	8	5.72

### 2.5. Photocatalytic Activity

**Figure 7:**
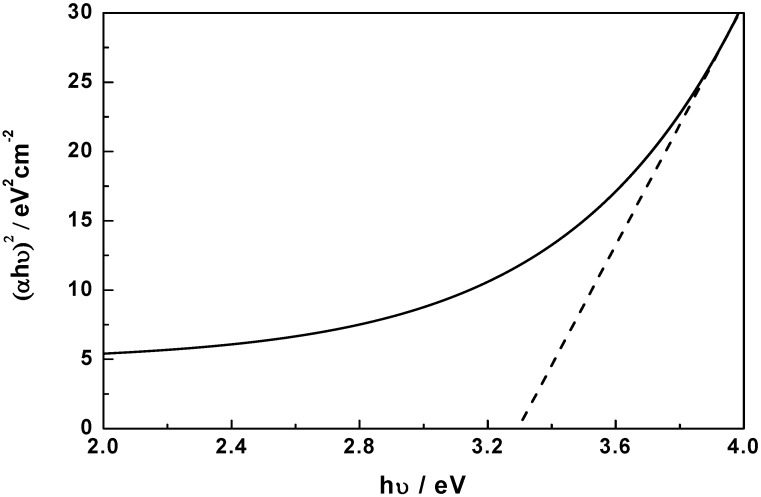
Evaluation of the band gap of flower-like NiO by the extrapolation of (*αhν*)^2^ *versus hν* curve.

**Figure 8:**
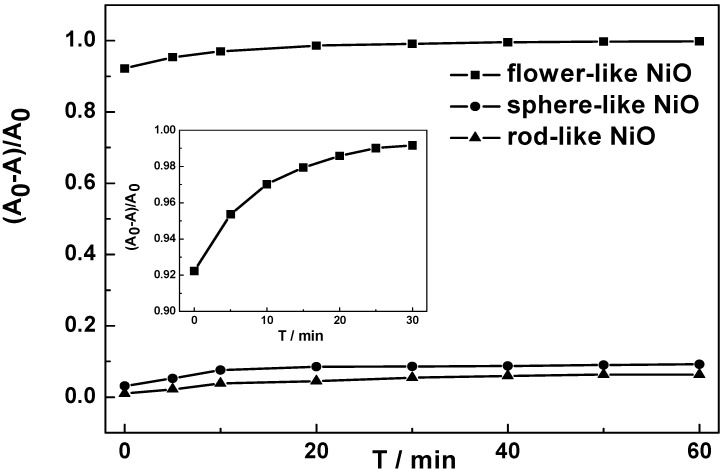
Degradation rates of MO at different intervals in the presence of NiO samples with different morphologies. The inset is the local magnification pattern of degradation curve in the presence of flower-like NiO.

Evaluating the UV-vis absorption spectra of the sample, the direct band gap of flower-like NiO obtained by extrapolation of the (*αhν*)^2^ *versus hν* curve is about 3.30 eV (Figure 7). Such a value is a negative shift to that of bulk NiO (3.5 eV) [22] and indicates photocatalytic performance, which could be ascribed to the special pore-wall structures. The photocatalytic activity of the flower-like NiO hierarchical structures was demonstrated by the degradation of MO in the aqueous solution under UV light irradiation. Before the irradiation, the NiO samples with different morphologies were kept in the MO solution of 20 mg·L^−1^ for 10 min to reach the adsorption/desorption equilibrium of MO molecules on samples. Figure 8 shows the effect of irradiation time on degradation rates of MO in the presence of flower-like, sphere-like and rod-like NiO samples, respectively. It can be seen from the magnification curve of the flower-like NiO sample that the degradation is initially rapid, and at 10 min 97% of MO is already removed. A steady value is reached up to 30 min and the removal capacity is measured to be 99.8%, at which MO is almost completely removed. However, the degradation capacity of the sphere-like and rod-like NiO is only 9.2% and 6.3%, respectively. Therefore, the flower-like hierarchical structures with high surface areas and more pore-wall structures is more advantageous to remove pollutions from waste water.

## 3. Experimental

### 3.1. Preparation of NiO Samples

NiCl_2_·6H_2_O (0.4754 g, 2 mmol) was dissolved in the mixture solution of ethylene glycol (EG) (10 mL) and deionized water (10 mL) to form a clear light green solution. NaOAc (1.44 g) and polyethylene glycol 200 (PEG200, 0.4 g) were sequentially added to the above solution. After stirring for 30 min to get a clear light green solution, this solution was transferred into a Teflon-line stainless-steel autoclave (25 mL capacity) and heated to 140 °C for 8 h. After the reaction, the autoclave was allowed to naturally cool to room temperature. The green products collected were rinsed with ethanol eight times, and then dried at 80 °C for 6 h, which was the intermediate product of NiO. Through the heat treatment at 350 °C for 2 h, NiO hierarchical structures with flower-like morphologies were formed.

For the preparation of sphere-like NiO, NiCl_2_·6H_2_O (0.7604 g, 3.2 mmol), polyvinylpyrrolidone (PVP, 0.2656 g) and hydrazine hydrate (1.6 mL) were first dispersed in EG (20 mL) under continuous stirring. Then this mixture solution was transferred to a Teflon-line stainless-steel autoclave (25 mL capacity) and heated to 190 °C for 8 h. The following experimental procedures were the same to those of flower-like NiO.

The fabrication of the rod-like NiO was similar to that of flower-like NiO, expect that EG (20 mL) was used to take the place of the mixture solution of EG (10 mL) and deionized water (10 mL) and the reaction temperature was set to 120 °C.

### 3.2. Characterization

The as-prepared samples were characterized by transmission electron microscopy (TEM, JEM-1010), scanning electron microscopy (SEM, JEOL JSM-6360LV SEM), X-ray diffraction (XRD, Shimadzu X-6000 X-ray diffraction, Cu Kα radiation), Fourier transform IR (FTIR, VECTOR 22 from BRUKER), and thermogravimetric analysis (TGA, LABSYS from SETERAM). Nitrogen adsorption and desorption isotherms were measured at 77 K using a Miromeritics ASAP2020M micropore analysis system. The specific surface area of the sample was detected by the Brunauer-Emmett-Teller (BET) method, and the average pore diameters were determined using the Barrett-Joyner-Halenda (BJH) method. UV-vis absorption spectra of the mixture of the sample and barium sulfate powder was measured on a SHIMADZU UV-2550 spectrophotometer.

### 3.3. Adsorption Activity

The adsorption activities of NiO samples were evaluated using various pollutants, such as methyl orange (MO), reactive red (RR), reactive brilliant blue (RBB) and reactive black (RB), as the model structures. The pollutant aqueous solution (5 mL, 20 mg·L^−1^) and NiO powder (10 mg) were mixed in a reactor under continuous stirring. At a defined time interval, the above suspension was separated by the centrifugal effect and the clear solution containing no adsorbed pollutant was analyzed on a HP8453 UV-vis absorption spectrophotometer.

### 3.4. Photocatalytic Property

The photocatalytic properties of the NiO samples with different morphologies were monitored by decomposing MO at room temperature. At first the sample (10 mg) was dispersed in the MO solution (5 mL 20 mg·L^−1^) in a quartz tubular container without light irradiation. After stirred for 10 min, the mixture was further stirred in front of a 500-W mercury lamp and cooled by quickly running water to avoid additional heating effects. The concentration of MO was tested by HP8453 UV-vis absorption spectrophotometer.

## 4. Conclusions

Flower-like NiO hierarchical structures made up of nanosheets have been successfully fabricated by a facile two-step and template-free method. Adjusting the reaction temperature allowed selective control of the preparation of flower-like hierarchical and sheet-like structures of NiO. A possible mechanism of formation of flower-like NiO deduced by investigating the effect of the reaction time on the morphologies of the samples is suggested. The flower-like hierarchical morphology provides a large specific surface area and bimodal mesopore distribution. Compared with the sphere-like and rod-like NiO, the flower-like NiO samples show the excellent and rapid adsorption and photocatalytic performance towards various pollutants, indicating the potential applications of the as-obtained products in water treatment.
